# Transfer Hydrogenation Employing Ethylene Diamine Bisborane in Water and Pd- and Ru-Nanoparticles in Ionic Liquids

**DOI:** 10.3390/molecules200917058

**Published:** 2015-09-17

**Authors:** Sebastian Sahler, Martin Scott, Christian Gedig, Martin H. G. Prechtl

**Affiliations:** Department of Chemistry, University of Cologne, Greinstrasse 6, 50939 Cologne, Germany; E-Mails: ssahler@hiden.co.uk (S.S.); scott@itmc.rwth-aachen.de (M.S.); cgedig@smail.uni-koeln.de (C.G.)

**Keywords:** ruthenium, palladium, transfer-hydrogenation, ethylene diamine bisborane, ammonia borane, EDAB, reducing, carbonyl

## Abstract

Herein we demonstrate the use of ethylenediamine bisborane (EDAB) as a suitable hydrogen source for transfer hydrogenation reactions on C-C double bonds mediated by metal nanoparticles. Moreover, EDAB also acts as a reducing agent for carbonyl functionalities in water under metal-free conditions.

## 1. Introduction

Hydrogenation is one of the most important chemical transformations used in academia and industry and has received notable attention in the past century [1,2,3,4,5,6,7]. It can be performed via transition metal-catalyzed activation of molecular hydrogen and is used in large-scale applications such as hydrocracking [8,9] and the Haber-Bosch-Process [10] or in medium- to lab-scale applications for the synthesis of fine and special chemicals [1,2,3,11]. However, as the use of molecular hydrogen often requires harsh reaction conditions [12] and the regio- and stereo-selectivity are challenging to control [4,5,7,13], transfer hydrogenation has evolved as useful tool for specific and highly selective hydrogenation under mild conditions [3,4,7,12,14,15]. Specifically, the use of alcohols such as ethanol, isopropanol, and glycerol [4,7,16,17] and also compounds like formic acid [18] or Hantzsch’s ester [19] have been used as suitable hydrogen sources in transfer hydrogenation reactions. Many of these transformations can be performed by using homogenous catalysis with transition metal complexes based on iron, palladium, ruthenium, or rhodium [4,7,15,17,20,21]. In contrast, the use of non-metal based organo-catalysts has also been shown and several prolin-based catalysts are known [22,23]. A major drawback of using alcohols or other carbon-based compounds in transfer hydrogenation lies in the comparable low mass fraction of the stored hydrogen volume-to-mass ratio. For example, isopropanol delivers only one equivalent (3.3 wt-% H_2_) and formic acid (4.4 wt-% H_2_) as well. Therefore, hydrogen-rich sources for transfer hydrogenation reactions are desirable. On the other hand, amine boranes such as ammonia borane (AB) (NH_3_BH_3_) [24], methyl amine borane [24,25] (MAB), and ethylene diamine bisborane [24,26] (EDAB) have received increasing attention as hydrogen storage materials owing their hydrogen content. The mass fraction of stored hydrogen is comparably high and the compounds are easily obtained [24]. MAB releases a mass fraction of 9% H_2_ [25,27], EDAB a mass fraction of 10% [26], and AB a mass fraction of up to 16% H_2_ at a maximum temperature of 200 °C [24]. Amine boranes have not only been used as possible hydrogen storage material. Additionally, these non-toxic and water-soluble compounds have shown successful use as hydrogen sources for transfer hydrogenation reactions. AB in particular has been used elaborately for the reduction of C=C [28], C=O [29], C=N [13,30], or NO_2_ [13], thereby showing chemo-selectivity [13]. However, for other amine boranes, the literature is scant and little is known about chemo-, regio-, and stereo-selectivity. Our group has investigated the liberation of H_2_ from EDAB in ionic liquid media as a potential hydrogen storage material [26,31]. Following these studies, we focus on the use of EDAB as a possible hydrogen source for transfer hydrogenation reactions.

Referring to the ongoing debate of implementing greener processes in chemistry [32,33], but also regarding excellent behavior in catalytic conversions [34], the use of tailor-made ionic liquid media as reaction mediums has revealed encouraging effects for dehydrogenation reactions with amine boranes [26,31]. The incorporation of homogeneously dispersed metal nanoparticles as highly potent catalysts has also been demonstrated [31,35,36,37,38,39,40,41]. Furthermore, the use of nanoparticles as catalysts for the hydrolytic dehydrogenation of AB has been shown [42,43,44,45]. Additionally, EDAB undergoes hydrolytic dehydrogenation with elevated hydrogen yields compared to the reaction in common organic solvents if ionic liquids (ILs) are implemented as reaction medium. Subsequently, transition metal nanoparticles have been used as catalysts in transfer hydrogenation reactions, reducing C=C [46] or C=O [47] functionalities. However, to the best of our knowledge, the use of nanoparticles as transfer hydrogenation catalysts in ILs as reaction media has not yet been reported. Furthermore, the use of EDAB as a suitable hydrogen source in transfer hydrogenation reactions is also unknown.

## 2. Results and Discussion

**Scheme 1:**
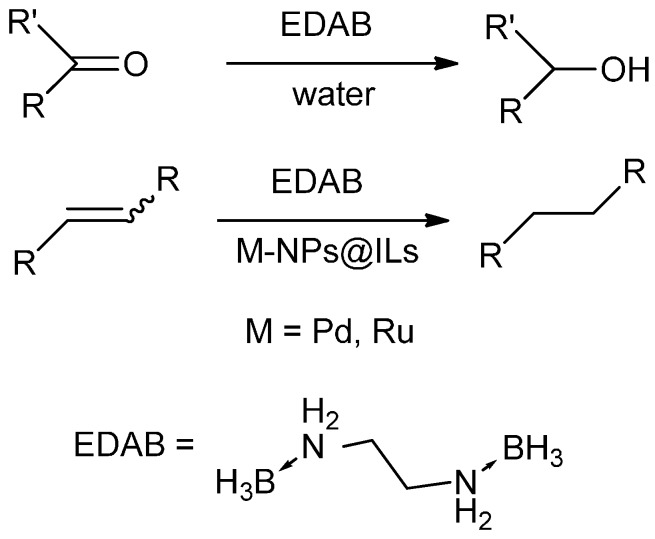
EDAB as a hydrogen source for the reduction of carbonyl functionalities and unsaturated carbon-carbon bonds.

Herein we present the use of EDAB as a hydrogen source as well as the use of Pd- and Ru-NPs (NPs: nanoparticles) as suitable transfer hydrogenation catalysts for the chemo-selective hydrogenation of carbonyl functionalities and for the reduction of unsaturated carbon-carbon bonds (Scheme 1).

The application of amine borane adducts as highly active bench-top reducing agents has long been known [48,49], but has fallen out of favor, and recently it has been demonstrated that they are suitable for safe reduction in pure water [50]. The reduction of carbonyl groups in different chemical surroundings is possible employing EDAB at mild temperatures and water as the solvent (Table 1).

Cyclohexanone (Table 1, entry 1) is quantitatively reduced by EDAB. The reduction of other simple carbonyl compounds like acetophenone (Table 1, entry 2), cyclohex-2-enone (Table 1, entry 3), and benzaldehyde (Table 1, entry 4) succeeds with moderate yields. While the aromatic substrates remain intact, the reduction of α, β-unsaturated cyclohex-2-enone yields a mixture of the unsaturated cyclohex-2-enol (50% yield) and the saturated cyclohexanol (13% yield). Interestingly, the reduction of cinnamic aldehyde (Table 1, entry 5) yields solely the unsaturated cinnamic alcohol. The double bond remains unchanged, probably due to the stabilizing effect of the conjugated aromatic system. The comparison of the aliphatic carbonyl substrates octanal (Table 1, entry 6) and octan-3-one (Table 1, entry 7) reveals that aldehydes are by far more reactive toward EDAB than similar ketones. While octanal is reduced in 33%–38% yield after 1 h, only 9% of octan-3-one reacts in the same time. The yield can be notably enhanced by prolonging the reaction time to 24 h (53%). The direct comparison between octanal and 3-octanone showed that 38% 1-octanol and 9% 3-octanol are produced. A similar behavior is observed when benzaldehyde and acetophenone are reduced in direct comparison: 93% phenylmethanol and 68% 1-phenylethanol are produced. These experiments indicate that the reduction of aldehydes is faster than ketones under the given conditions.

The reduction of benzophenone yields diphenylmethanol in moderate amounts (48%–53%; Table 1, entry 8), while benzoquinone does not react under the given conditions (Table 1, entry 9). In contrast to the reduction of octan-2-one, the reduction of octan-2, 3-dione (Table 1, entry 10) yields moderate octan-2, 3-diol amounts (37%–42%). The improved reactivity might be related to the superior solubility of the dione in comparison to the single ketone. Reduction of 5-hydroxymethylfurfural (HMF) also shows only a slow conversion into furandimethanol (Table 1, entry 11; 10%). Several other functional groups are not reduced by EDAB: alkenes, aromatic double bonds, nitro groups, lactames, esters, ethers, and acids remain inert, making EDAB highly chemo-selective for the reduction of aldehydes and ketones (Table 1, entry 12–16). The main influences on the yields of the reductions appear to be the solubility and the steric hindrance of the substrate.

**Table 1:** Metal-free reductions using EDAB at 80 °C in H_2_O.

#	Reaction	Yield ^a^ (%)
1	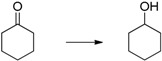	99
2	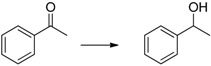	68
3	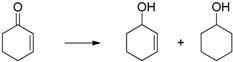	50 (+13) ^b^
4	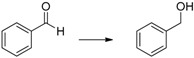	75–93
5	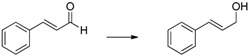	50–72
6	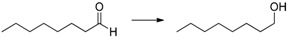	33–38
7	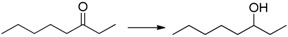	9 (53) ^c^
8	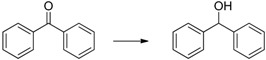	48–53
9	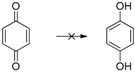	0
10	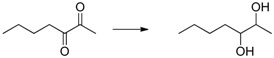	37–42
11	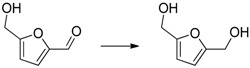	10
12	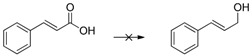	0
13	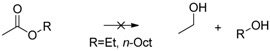	0
14	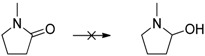	0
15	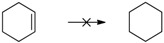	0
16	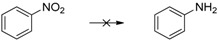	0

^a^ The yield was determined by ^1^H-NMR using Si_2_Me_6_ as internal standard. Each reaction was performed at least twice with a reaction time of 1 h; ^b^ 13% of cyclohexanol was found; ^c^ after 60 min and 24 h, respectively.

In comparison to the reduction employing ammonia borane (AB) or methyl amine borane (MAB) [50,51], reduction by EDAB is slightly slower, even at higher temperatures. The scope of substrates is very similar for all three reducing agents; a plethora of different carbonyl compounds are susceptible to reduction, leaving nearly all other functional groups intact.

Besides the reduction of carbonyl compounds with neat EDAB in water, the application of metal nanoparticles as catalysts broadens the substrate scope considerably. Ruthenium and palladium were chosen as catalysis. These metals in particular are known to be active in hydrogenation and dehydrogenation reactions in ionic liquid media [31,35,36,37,39,41]. Additionally, ionic liquids were employed due to their stabilizing effect on the nanoparticles as well as the ionic solvent properties, which provide a good solubilization of the substrates. We evaluated the well-characterized nanoparticle catalysts Ru@[BMMIM][NTf_2_] (BMMIM: 1-*n*-butyl-2,3-dimethylimidazolium) and Pd@[(BCN)MIM][NTf_2_] ((BCN)MIM: 1-butyronitrile-3-methylimidazolium) [35,39] for transfer hydrogenation with EDAB as the hydrogen source. The results for the ruthenium-catalyzed reactions are listed in Table 2.

**Table 2:** Catalyzed transfer hydrogenations with EDAB and Ru@[BMMIM][NTf_2_] at 70 °C after 18–24 h.

#	Substrate	[%] ^a^	[%] Overall	TON ^b^
1	cyclohexene	25	25	72 (137)
2	1,5-cyclooctadiene	55 (5)	60	133 (253)
3	benzonitrile	0.0	0	0.0
4	nitrobenzene	0.0 (17)	17	108 (205)
5	phenylacetylene	8.1 (7.7)	16	50 (95)
6	diphenylacetylene	22 (5)	27	45 (86)
7	styrene	45	45	127 (241)
8	squalene	0 (12)	12	51 (97)
9	1-octene	33	33	92 (176)
10	2-octene	41	41	112 (214)
11	1-octyne	21 (50)	72	253 (482)
12	4-octyne	31 (14)	45	129 (245)
13	1,3-cyclohexadiene	20 (6)	26	69 (131)
14	1,4-cyclohexadiene	22 (3)	26	59 (112)

^a^ In case of triple bonds the product of single hydrogenation is the *Z*-isomer, exclusively; ^b^ The TON in brackets is considered with the *magic number approach*, with a particle size of 2.0 nm [35,37] which corresponds to 52.6% surface atoms. Each reaction has been performed at least twice.

Among the tested compounds, simple alkenes and alkynes showed the highest conversion upon use of the Ru@[BMMIM][NTf2] system. The hydrogenation of cyclohexene succeeds with mediocre TONs (Table 2; entry 1). 1,5-cyclooctadiene can be hydrogenated with moderate TONs of 253 considering only available surface atoms, where the single hydrogenation is strongly favoured over the full hydrogenation (Table 2; entry 2; 55%:5%). The hydrogenation of 1-octyne shows an inverse selectivity (21%:50%) and also the highest activity observed overall (Table 2, entry 11; TON 482). In comparison 4-octyne is hydrogenated, but to a smaller extent (TON 245) and less selective (Table 2, entry 12; 31%:14%), probably due to the higher steric hindrance. The alkynes phenylacetylene and diphenylacetylene are slowly converted with TONs of 50 and 45, respectively, and selective towards the corresponding alkenes are observed (Table 2, entries 5–6). In case of alkenes, the highest conversion is observed with styrene (Table 2, entry 7; TON 241). Squalene, 1-octene, 2-octene as well as 1, 3- and 1,4-cyclohexadiene are hydrogenated slightly slower (Table 2; entries 8–10 and 13–14). While benzonitrile (Table 2, entry 3) remains unchanged, nitrobenzene (Table 2, entry 4) can be converted to aniline with a TON of 205. No hydrogenation of arenes was observed for these functionalised arenes, which stays in agreement with earlier results that employed elemental hydrogen as the reducing agent for functionalised and unfunctionalized arenes [35,36,37,41].

Moreover, palladium nanoparticles in the IL [(BCN)MIM][NTf_2_] have been evaluated for transfer hydrogenation from EDAB to organic substrates containing alkynes and alkenes (Table 3). 

**Table 3:** Catalyzed transfer hydrogenations with EDAB and Pd@[(BCN)MIM][NTf_2_] at 70 °C after 18–24 h.

#	Substrate	[%] ^a^	[%] Overall	TON ^b^
1	phenylacetylene	9.5 (8.1)	18	122 (665)
2	diphenylacetylene	54–69 (1–9)	64–71	186–216 (1020–1182)
3	1-octyne	28 (32)	59	449 (2453)
4	4-octyne	54	11	400 (2186)
5	1-octene	45	45	255 (1395)
6	2-octene	37	37	245 (1337)
7	styrene	47	47	316 (1727)
8	cyclohexene	34	34	199 (1090)
9	benzonitrile	0	0	0.0
10	squalene	traces	n.d.	n.d.
11	1,3-cyclohexadiene	42 (0.2)	42	197 (1075)
12	1,4-cyclohexadiene	43 (3)	46	229 (1253)
13	1,5-cyclooctadiene	4	0.2	19 (104)

^a^ In case of triple bonds the product of single hydrogenation is the *Z*-isomer, exclusively; ^b^ The TON (turnover number) in brackets is considered with the m*agic number approach*, with a particle size of 7.3 nm [39], which corresponds to 18.3% surface atoms. Each reaction has been performed at least twice. n.d.: not determined.

The hydrogenation of phenylacetylene (Table 3, entry 1) proceeds with overall small yields and low selectivity. Interestingly, diphenylacetylene is hydrogenated somewhat faster, despite the higher steric demand (Table 3, entry 2). A strong selectivity towards the single hydrogenated *Z*-alkene is observed (69%:1%). The hydrogenation of 1- and 4-octyne (Table 3, entries 3–4) succeeds with TONs of 2453 and 2186, respectively. Selectivity differs strongly among these substrates: the fully hydrogenated product of 1-octyne is found in higher abundance (32% of 59% overall yield) than that of 4-octyne (entry 4, 11% of 65% overall yield). Simple alkenes, *i.e.*, 1-octene, 2-octene, styrene, and cyclohexene (Table 3, entries 5–8), are hydrogenated with moderate TONs of 1090–1727. No hydrogenation of arenes is observed. Similarly to the reaction employing the previously described Ru-system, nitriles are not hydrogenated (Table 3, entry 9). Squalene (Table 3, entry 10) yields only product traces and the reduction of nitrobenzene proceeds uncontrolled and was not further examined. The dienes 1,3- and 1,4-cyclohexadiene (CHD) (Table 3, entries 11–12; CHD) can be hydrogenated with similar TONs (1075–1253) and selectivities (1,3-CHD: 0.2% cyclohexane of 42% overall yield and 1,4-CHD: 3% cyclohexane of 46% overall yield). Hydrogenation of 1,5-cyclooctadiene (Table 3, entry 13) yields only minor amounts of product (entry 13, 4% cyclooctene of 4.2% overall yield). Conclusively, the substrates accessible to transfer-hydrogenation with Pd@[(CN)BMIM][NTf_2_] using EDAB are very similar to those of the Ru-system previously described. In most cases, the Pd-system shows a higher activity and a better selectivity towards the partial hydrogenated product (Table 4). In the reduction of 1,5-cyclooctadiene and squalene, the Ru-system showed a considerably higher activity. The reason for these exemptions is yet to be found.

**Table 4:** Comparison between Ru-NPs and Pd-NPs.

	Substrate	Sing. Transf.	Mult. Transf.	Ru-NP@(IL-1)			Pd‑NP@(24) or @(IL-2)		
				TON	Conv. [%]		TON	Conv. [%]	
					Sing.	Mult.		Sing.	Mult.
1	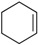	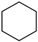	-	211	25	-	1108	2.70	34
2	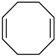	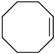	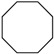	392	55	5	106	4 ^a^	0
3	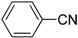	-	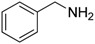	0	-	0	0	-	0
4	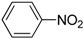	-	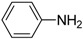	318	-	17	deflagration		
5	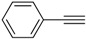	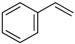	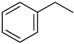	147	8	8	676	10	8
6	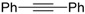	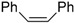	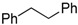	133	22	5	1202	69	2
7	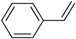	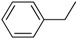	-	372	45	-	1756	47	-
8	Squalene	-	Squalane	151	-	12	n.d.	-	traces
9	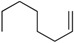	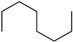	-	272	33	-	1418	45	-
10	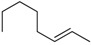	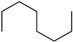	-	331	41	-	1359	37	-
11	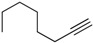	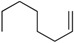	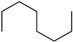	745	21	50	2494	28	32
12	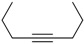	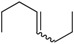	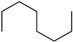	0	31	14	2223	54	10
13	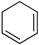	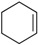	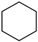	380	20	6	1092	42	0
14	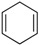	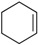	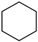	202	22	3	1274	43	0

^a^ [(BCN)M2Im][NTf2] was used.

## 3. Experimental Section

### 3.1. General

All commercial chemicals and solvents were purchased from Sigma-Aldrich (St. Louis, MO, USA), Merck (Kenilworth, NJ, USA) and Acros (Waltham, MA, USA) and used as received. Ethylene diamine bisborane (EDAB) [26,27,31], all used ionic liquids [39,52], ruthenium nanoparticles [37], and palladium nanoparticles [39] were prepared using previously published procedures. Nuclear magnetic resonance spectroscopy was performed at 300 K using a Bruker Avance II 300 or a Bruker Avance II 200 by Bruker (Billerica, MA, USA) at given frequency: AvanceII 300 (^1^H (300.1 MHz), ^13^C (75.5 MHz), ^11^B (96.2 MHz), ^19^F (282.2 MHZ)), and AvanceII 200 (^1^H (200.1 MHz) ^13^C (50.0 MHz)). If not otherwise indicated, all measurements were proton broad band-decoupled. The relative chemical shift δ is referenced in the remaining signal of the given deuterated solvent for ^1^H and ^13^C spectra. For ^11^B spectra, BF_3_-diethylether complex was added as standard in sealed glass ampules. For ^19^F spectra, trifluoromethane (δ = 0 ppm) was used as internal standard, respectively. All chemical shifts are given in *parts per million* (ppm)*.* When possible, the multiplicity of each spectrum is assigned using the following abbreviations: s: singlet, d: doublet, t: triplet, q: quartet quint: quintet, sext: sextet, sept: septet, m: multiplet, br: broad signal, and mc: multiplet, centred. GC-MS was performed on an Agilent GC-system series 6890 by Agilent using a capillary column HP5MS–0.25 μm of 30 m length and 0.32 mm in diameter by J & W (J & W Scientific, branch of Agilent, Santa Clara, CA, USA). The used temperature program 50–300 M had a temperature increase of 10 °C/min with a starting temperature of 50 °C, an ending temperature of 300 °C, and a run time of 5 min. Mass detection was carried out on an Agilent 5973 Network Mass Selective Detector by Agilent. Ionization was achieved by electron impact ionization (EI) with an ionization potential of 70 eV.

### 3.2. Experimental Protocols

#### 3.2.1. Nanoparticle Synthesis

##### Ruthenium Nanoparticle Synthesis [35,37]

The synthesis of Ru-NPs was carried out according to literature-reported procedures [35,37]. Under glove box conditions, a screw neck vial was loaded with approximately 5–15 mg of bis(2-methylallyl)(1,5-cyclooctadiene)ruthenium(II) and 0.65–1.45 mg of ionic liquid [BMMIM][NTf_2_] and sealed. The reaction mixture was stirred 18 h at 75 °C to yield a brown to dark brown but clear suspension. The obtained particle size averaged 2.0 nm with a size distribution of ± 0.5 nm [35,37].

##### Palladium Nanoparticle Synthesis [39]

The synthesis of Pd-NPs was carried out according to a literature-reported procedure [39]. A screw neck vial was loaded with approximately 3 mg of palladium(II)acetate and 600 mg of ionic liquid [(BCN)MIM][NTf_2_] and sealed. The reaction mixture was stirred 3 h at 130 °C to yield an orange but clear suspension. The obtained particle size averaged 7.1 nm with a size distribution of ± 2.2 nm [39].

#### 3.2.2. Metal-Free Transfer Hydrogenation in Water

**Exemplary procedure (Table 1; entry 2):** A vial was charged with 2.3 mmol acetophenone (Table 1) and 5 mL demineralized water. After adding 0.5 mmol of EDAB (four H_2_ equivalents), the vial was sealed, placed in an aluminum heating block at 80 °C, and the mixture was stirred for the given time (Table 1). Subsequently, the aqueous suspension was cooled down and extracted three times with 5 mL Et_2_O. The organic layers were combined and the solvent was removed under reduced pressure. The product yield was determined by NMR spectroscopy using a defined amount of Si_2_Me_6_ as internal standard.

#### 3.2.3. Transfer Hydrogenations in Ionic Liquids with Metal Nanoparticles 

**Exemplary procedure (Table 2; entry 1):** In a typical transfer hydrogenation experiment, a vial was charged with the ruthenium nanocatalyst mixture of previously synthesized ruthenium nanoparticles (3 mg Ru) in ionic liquid (1.28 g; Ru@BMMIM][NTf_2_]) [37] and 9 mmol cyclohexene. Subsequently, 1.7 mmol EDAB was added and the vial was sealed. The reaction mixture was stirred overnight at 70 °C and then cooled to room temperature. A defined amount of CDCl_3_ as solvent and hexamethyl disilane (HMDS; Si_2_Me_6_) as internal standard was added to the mixture and homogenized. A sample was taken and the product yield was determined by NMR spectroscopy (Table 2 and Table 3). The Pd-catalyzed reactions used 600 mg [(CN)BMIM][NTf_2_] and 0.5 mg Pd nanoparticles prepared as previously published [39].

## 4. Conclusions

In summary, ethylenediamine bisborane (EDAB) is a suitable hydrogen source for transfer hydrogenation reactions on C-C double bonds mediated by metal nanoparticles. Several organic model compounds could be shown to undergo reduction in the presence of the Ru-system as well as the Pd-system. In most cases, the latter showed considerably higher activity. The single hydrogenation of triple bonds yields only *Z*-isomeric compounds, as expected in a heterogeneous catalytic reaction. C-N triple bonds (nitriles) are not susceptible to the reduction. C-O double bonds can be reduced without the presence of metal particles in water. C-C double bonds can be readily reduced, while aromatic double bonds remain inert. In future investigations, the selectivity, time-resolved studies, and solvent effects will be addressed. The reduction with this air- and moisture-stable amine borane adduct could complement the synthetically interested chemist's toolkit.
